# Association between Social Isolation and Total Mortality after the Great East Japan Earthquake in Iwate Prefecture: Findings from the TMM CommCohort Study

**DOI:** 10.3390/ijerph19074343

**Published:** 2022-04-05

**Authors:** Yuka Kotozaki, Kozo Tanno, Kiyomi Sakata, Kotaro Otsuka, Ryohei Sasaki, Nobuyuki Takanashi, Mamoru Satoh, Atsushi Shimizu, Makoto Sasaki

**Affiliations:** 1 Iwate Tohoku Medical Megabank Organization, Iwate Medical University, Morioka 028-3694, Iwate, Japan; ktanno@iwate-med.ac.jp (K.T.); ksakata@iwate-med.ac.jp (K.S.); kotaro29@df6.so-net.ne.jp (K.O.); pears_takanashi@ybb.ne.jp (N.T.); satohm@iwate-med.ac.jp (M.S.); ashimizu@iwate-med.ac.jp (A.S.); masasaki@iwate-med.ac.jp (M.S.); 2 Department of Hygiene and Preventive Medicine, School of Medicine, Iwate Medical University, Morioka 028-3694, Iwate, Japan; 3 Department of Neuropsychiatry, School of Medicine, Iwate Medical University, Morioka 028-3694, Iwate, Japan; 4 Division of Physical Education, Department of Human Sciences, Center for Liberal Arts and Sciences, Iwate Medical University, Morioka 028-3694, Iwate, Japan; ryou-hei-1115@topaz.ocn.ne.jp; 5 Division of Biomedical Information Analysis, Institute for Biomedical Sciences, Iwate Medical University, Morioka 028-3694, Iwate, Japan; 6 Division of Ultrahigh Field MRI, Institute for Biomedical Sciences, Iwate Medical University, Morioka 028-3694, Iwate, Japan

**Keywords:** social isolation, mortality, cohort study, the Great East Japan Earthquake, house damage, the death of family member

## Abstract

This study aimed to investigate whether social isolation is associated with mortality, together with the effect of the Great East Japan Earthquake on mortality, due to the social isolation of community residents living in the affected areas, using data from the Tohoku Medical Megabank Project Community-Based Cohort Study. A total of 22,933 participants (8059 men and 14,874 women), who were free from cancer and cardiovascular disease, were followed up with death as an endpoint for five years. Social isolation was assessed using the Lubben Social Network Scale (cut-off, 11/12). Using Cox proportional hazards models, hazard ratios (HRs) of total mortality and 95% confidence intervals (CIs) associated with social isolation (no isolation as the reference) were estimated. The latter was significantly associated with an increased risk of total mortality (1.38 (1.04–1.83) in men and 1.49 (1.02–2.19) in women). Moreover, among those with social isolation, the risk of mortality was significantly higher, especially for women with house damage and men who had experienced a death in the family. The disaster may have raised the risk of mortality due to social isolation.

## 1. Introduction

The Great East Japan Earthquake (GEJE) of 2011 caused catastrophic damage, specifically, in the coastal areas of Iwate and Miyagi prefectures near the epicenter [1,2]. With a magnitude of 9.0, it was the strongest earthquake ever recorded in Japan; the maximum intensity of the earthquake was 7, and a tsunami of more than 9.3 m hit the area, with a maximum run-up height of 40 m [2]. According to the U.S. Geological Survey (USGS), it was the fourth largest earthquake in the world since 1900 [3]. The tsunami inundated a total area of 561 square kilometers, mainly on the Pacific side of Tohoku [4]. The death toll from the GEJE stands at 15,900, and 2523 people are missing, as of 1 March 2022 [1]. Immediately after the earthquake, many people took refuge in evacuation centers and shelters. At its peak, on the third day after the earthquake, more than 400,000 people were evacuated in the three affected prefectures of Iwate, Miyagi, and Fukushima [5]. Due to the damage to houses and injuries to people caused by the GEJE, many lost their social ties, and there were concerns about the social isolation of the residents in the affected areas.

Social isolation is an objective and quantifiable outcome of reduced social network size [6,7]. Social networks are important for the quality of friendships and family ties [8]. Previous studies showed that people who are socially isolated have an increased risk of developing cardiovascular disease [9,10], depressive symptoms [11,12], and cognitive decline [13,14]. Moreover, it has been suggested that social isolation can affect mortality [8,15].

Social isolation facilitates the immediate and delayed effects of disaster stress [16]. Epidemiological studies of Japanese people reported an increase in the social isolation rate after the GEJE [17]. There have been several reports on the relationship between social isolation and depressive symptoms after the GEJE [17,18,19]. A survey of 860 residents over the age of 65 in Iwanuma City, Miyagi Prefecture, reported that participants without any social interactions had higher mortality rates in the 38-month interval after the GEJE [20]. However, no study has evaluated whether the severity of house damage and the death of family members due to a large-scale natural disaster affect the association between social isolation and mortality.

Therefore, this study aimed to investigate whether social isolation is associated with an increased risk of total mortality, as well as the effect of the disaster on total mortality, due to the social isolation among community residents living in areas affected by the GEJE.

## 2. Materials and Methods

### 2.1. Study Population

This study is a part of the Tohoku Medical Megabank Project Community-Based Cohort Study (TMM CommCohort Study) [21,22]. Recruitment of participants was conducted at municipal health check-ups and our facility. The inclusion criteria were persons aged 20 years or older who were registered in the basic resident register of all municipalities in Iwate Prefecture at the time of enrollment. The baseline data were collected between 2013 and 2016.

We followed 32,320 of the 32,919 participants who participated in the baseline study, excluding the six dual enrollees and 593 who withdrew consent. Among these, we excluded 868 participants who did not return the questionnaire; 4162 who had a history of cancer, cardiovascular disease, or stroke to avoid the effect of reverse causation; 2270 who had missing data on the Lubben Social Network Scale (LSNS-6); and 2087 who had missing covariate variable data. Finally, we analyzed 22,933 participants (8059 men and 14,874 women; mean age at baseline survey 58.8 ± 11.8 years). The Ethics Committee of Iwate Medical University (first approval: HG H25-2; most recent approval: HG 2018-004) approved all the study procedures.

### 2.2. Measurements

#### 2.2.1. Social Isolation

Social isolation was assessed using the LSNS-6 [7,23]. It comprises six items on social connections (three questions on family ties and three questions on friendship ties). Each item is rated on a six-point Likert scale. The LSNS-6 scores ranged from 0 to 30. The reliability and validity of the Japanese version have been confirmed [24]. Social isolation was defined as a score <12 [7,24].

#### 2.2.2. House Damage and the Death of Family Members Due to the GEJE

We used six options to assess house damage caused by the GEJE: (1) completely damaged (including all outflows), (2) seriously damaged, (3) half-damaged, (4) partially damaged, (5) no damage, and (6) non-residence. We further classified these options as damaged (options 1 to 4) or undamaged (options 5 and 6). Those with damage were likely to be evacuated to temporary housing, which may have excluded them from their community. Even if some communities move to temporary accommodating areas, maintenance social contact could be reduced. Participants responded yes or no to inquiries about the death of family members due to the GEJE.

#### 2.2.3. Follow-Up Survey

The follow-up period for the survival analysis was until 31 December 2019. We identified deaths and move-outs by annually electronically collating with the municipal basic resident register or by requesting a resident certificate for all the participants, at an approximately similar time. The death referred to the date of death, and move-out to that of move-out. For those who withdrew consent for follow-up, it was terminated at the date of withdrawal of consent. For those who had no events, the follow-up period was until the last day of follow-up. There were 36 participants whose resident certificates had not been issued or whose whereabouts could not be confirmed, even after requesting a resident certificate. Therefore, they could not be followed up and were excluded from the analysis.

#### 2.2.4. Covariates

Demographic characteristics were recorded in the questionnaire, except for body mass index (BMI). BMI was calculated by measuring body weight and height at the site of health check-ups and our facility. These were included in the analysis as covariates: age (continuous); inland or coastal area (with inland area indicating that the participant’s municipality was not shown on a map as bordering a sea, and coastal area indicating that it was shown on a map as bordering a sea); education level (junior high school, high school, college, university or higher); marital status (unmarried or married); the number of household members (living alone or ≥2); work status (unemployed or employed); smoking habits (non-smoker or current smoker); drinking habits (non-drinker or current drinker); medical history (hypertension, diabetes mellitus, and hyperlipidemia); BMI (<18.5, 18.5 to <25, or ≥25 kg/m^2^); depressive symptoms; insomnia; and social capital. Depressive symptoms were determined by a score of ≥16 on the Center for Epidemiological Studies Depressive Scale (CES-D) [25,26]. Insomnia was defined as a score of ≥6 on the Athens Insomnia Scale [27,28]. Social capital was determined using a score of <9 on the social capital scale [29].

### 2.3. Statistical Analysis

We analyzed the data from both sexes separately because there is a difference in the association between social isolation and mortality [30,31,32]. Continuous variables were summarized as mean and standard deviation, and categorical variables as percentages. The χ^2^ test for categorical variables and Student’s t-test for continuous variables were used to evaluate differences in characteristics. We used Cox proportional hazards regression models to estimate the hazard ratios (HRs) of total mortality and 95% confidence intervals (CIs) associated with social isolation, using the no isolation group as the reference category. The proportionality assumptions of the hazard by the LSNS-6 were verified using log-minus-log curves. Stratified analyses were performed. We calculated the HRs and 95% CIs for mortality according to social isolation by age group (<65 or ≥65 years of age). The model used for these analyses was the same as that used for the models calculated earlier. Moreover, to investigate the impact of house damage due to the GEJE and social isolation on mortality, we divided the participants into four groups according to the two factors and determined the HRs and 95% CIs for mortality in the three groups compared with the group without house damage or social isolation. Similarly, participants were classified into four groups based on the aforementioned factors, and the HRs and 95% CIs for mortality in the three groups were compared with the group without the death of family members or social isolation. Interaction terms were also considered in the stratified analysis.

Moreover, to avoid the possibility of reverse causalities, such as isolation due to the inability to leave the house because of illness, we performed the same analysis, excluding 41 participants who died within one year of the baseline survey.

All statistical analyses were conducted using SPSS version 25.0 for Windows (IBM, Tokyo, Japan). Statistical significance was set at *p* < 0.05.

## 3. Results

The characteristics of the participants, according to the presence or absence of social isolation, are shown in Table 1. In the overall study population, the prevalence of social isolation was 29.9% and 26.5% in men and women, respectively. In both sexes, compared with participants without social isolation, those with it were younger and more likely to be unmarried, have low exercise habits, have depressive symptoms, have insomnia, and lack social capital. In men, those with social isolation were more likely to be unemployed, live alone, and have hyperlipidemia, and less likely to be current drinkers than participants without social isolation. In women, those with social isolation were more likely to be current smokers and have low BMI, and less likely to be unemployed, have hypertension and hyperlipidemia than participants without social isolation.

During the follow-up period (a total of 115,624 person-years; mean follow-up period was 5.04 (SD 0.26) years), the number of deaths was 228 men and 133 women. The crude mortality (per 1000 person-years) was 5.10 without social isolation and 6.92 with it for men, and 1.68 without social isolation and 2.01 with it for women. The adjusted HRs (95% CIs) for mortality according to social isolation are presented in Table 2. Social isolation was significantly associated with an increased risk of mortality in both sexes after adjusting for all covariates (1.38 (1.04–1.83) in men and 1.49 (1.02–2.19) in women).

The adjusted HRs (95% CI) for mortality according to social isolation stratified by age group are shown in Table 3. There was no interaction between age and social isolation in either men or women. There was no interaction between age group and social isolation for both sexes (*p* = 0.660 in men and *p* = 0.416 in women).

The adjusted HRs (95% CIs) for mortality according to house damage and social isolation are shown in Table 4. In women, the HR was the highest for those with house damage and social isolation (HR (95% CI), 2.12 (1.17–3.83)). There was no interaction between house damage and social isolation for both sexes (*p* = 0.153 in men and *p* = 0.072 in women). The adjusted HRs (95% CIs) for mortality according to the presence or absence of death of family members caused by the GEJE and social isolation are shown in Table 5. The men showed that the HR was highest for those who experienced the death of family members or social isolation due to the GEJE (HR (95% CI), 1.69 (1.07–2.66)). There was no interaction between the death of family members and social isolation for both sexes (*p* = 0.080 in men and *p* = 0.125 in women).

Moreover, we performed the same analysis, excluding 41 participants who died within one year of the baseline survey. Social isolation was significantly associated with increased mortality risk in women after adjusting for all the covariates (Table S1). The adjusted HRs (95% CI) for mortality according to social isolation stratified by age group are shown in Table S2. There was no interaction between age group and social isolation in both sexes (*p* = 0.292 in men and *p* = 0.385 in women). The adjusted HRs (95% CIs) for mortality according to house damage and social isolation are shown in Table S3. Among women, the HR was highest for those with house damage and social isolation (HR (95% CI), 2.28 (1.23–4.21)). There was only an interaction between house damage and social isolation in women (*p* = 0.048). The adjusted HRs (95% CIs) for mortality according to the presence or absence of death of family members caused by the GEJE and social isolation are shown in Table S4. There was no interaction between the death of family members and social isolation regarding both sexes (*p* = 0.309 in men and *p* = 0.085 in women).

## 4. Discussion

We showed that socially isolated individuals had approximately twice the risk of total mortality compared with those who were not isolated regarding both sexes. Moreover, among those with social isolation, the risk of mortality was significantly higher, especially for women with house damage and men who had experienced a death in the family.

The mortality risk of social isolation has been consistently observed among diverse populations, regardless of gender, length of follow-up, and the region of residence [15,33,34,35]. Regarding the association between social isolation and mortality after a disaster, only one study has been conducted [20]. Aida et al. analyzed the risk factors for mortality up to 38 months after the GEJE separately from a survey of residents over the age of 65, and found that participants without social interactions had higher mortality rates. Our study focused on people aged 20 years and above, and the LSNS-6 was used as a scale to assess social isolation and total mortality due to social isolation by sex, and showed that it is associated with mortality, even if the follow-up period is more than five years, as well as that social isolation, severe house damage, and the death of family members caused by a large-scale natural disaster may, together, be associated with a higher mortality risk. The strength of our study is that it used a large enough sample size to investigate whether there is an association between social isolation and total mortality in both sexes and to examine the impact of disasters on the latter due to social isolation.

In the GEJE, due to house damage and other factors, the social ties that had been cultivated in the areas where people had lived were cut off, and many had to connect with those in the new areas where they had begun to live [36]. People who are prone to social isolation might feel more isolated due to the disaster, as they are placed in an environment where it is difficult to interact with those with which they used to have connections, or as a result of a sense of stagnation and decreased willingness to connect with others [37]. Moreover, continued social isolation makes it increasingly difficult to communicate with others, increases unresolved anxieties, worries in daily life, and might lead to mortality due to the health effects of continued physical inactivity [38].

Our study showed that socially isolated individuals aged over 65 years were significantly associated with mortality in both sexes after adjusting for all covariates. A study of 1270 outpatients reported that those who were older and socially isolated had a higher mortality risk [39]. Data from 6500 adults who participated in the English Longitudinal Study of Aging also reported that social isolation is associated with higher mortality in older men and women [8]. The elderly are more likely to become socially isolated than those in their working years, as they tend to interact less with others and have fewer household members [40]. Valtorta and Hanratty suggested that although the experience and consequences of isolation vary, for those made vulnerable by sickness or poverty, the impact is profound [40]. It is possible that the individuals who are more likely to be socially isolated are less likely to receive medical and social support, which may make them more likely to be exposed to mortality.

Our results showed that family death and social isolation were associated with death among men and house damage and social isolation were associated with death among women. Studies have reported that social isolation and mortality after a disaster are linked to house damage and the death of family members [8,15,20,34,41]. A study of the tsunami aftermath and recovery conducted after the 2004 Indian Ocean tsunami reported that people living near the heavily damaged coast had a higher mortality rate [41]. A survey conducted in Miyagi prefecture after the GEJE reported that the mortality rate was higher for people who lived near the coast, where most damage occurred during the earthquake [20]. Laugesen et al. reported that lack of a partner and social isolation were associated with the highest mortality [34]. As different combinations in men and women were associated with mortality, we considered that the following might be true: In Japan, men have spent their working lives focusing on one job and have few relationships in the community [42]. Men’s post-retirement relationships are often limited to their spouses and families. There is a lack of connection with people outside of the family. Therefore, the death of a family member due to the GEJE may make them more socially isolated; if there is no third party they can turn to when something goes wrong, they are not able to receive proper support in case of psychological or physical problems, which might lead to mortality. However, women are more likely to feel stressed about losing their homes and property during a disaster [43]. They tend to be more sentimental about the loss of important things associated with memories, while men tend to take a more realistic view [43]. This suggests that women who lost their homes might have felt the loss of their valued possessions for a long time. Moreover, if they are socially isolated, they may have to carry their feelings, which may affect their health and lead to mortality. The impact of disasters on the mortality of people susceptible to social isolation warrants attention. Social isolation is associated with mortality and public organizations provided support in various ways. However, strategies for providing effective support are lacking [15,44]. Previous studies have suggested that social interactions are protective against mortality [15,20,45], and there is a need to rethink the way we provide support for social isolation.

This study has several limitations. First, this target population may have had greater health awareness and better health status compared with the general population in the target area; thus, mortality may have been underestimated, and the generalization of results must be considered carefully. Second, there is the possibility of reversal of causality, such as isolation, due to the inability to leave the house because of illness. To eliminate this, we excluded deaths within one year, and the results were similar to the primary results. Therefore, we believe that our results do not indicate reverse causality. Third, there is the possibility of residual confounding. Fourth, no cause of death could be recorded because we did not have data on this. Fifth, because we do not have the data for prior to the GEJE, it is not possible to refer to what extent social isolation levels prior to the GEJE impacted disaster response preparedness, which would impact mortality rates. Finally, the level of social isolation and other measures at baseline was only measured once. Therefore, a single measurement of social isolation during the baseline survey may understate the relationship between social isolation and mortality, due to regression dilution effects [46]. Nevertheless, because our results show a significant association, we believe that they are robust. This study is significant because few known studies have reported an association between social isolation and mortality after an earthquake, using a population-based cohort study design and a large sample size.

## 5. Conclusions

Survivors of the GEJE in social isolation were more likely to have significant mortality than those without social isolation. We also found that the mortality risk among socially isolated people was increased by house damage and the death of family members due to the earthquake. Our results suggest that the association between social isolation and mortality risk is strengthened by house damage or the death of family members due to a large-scale disaster, and provides evidence to suggest that social support must be provided at the earliest possible stage to prevent the death of those who are socially isolated and have experienced house damage or the death of family members. Local governments must provide strategic social support to these people, in collaboration with relevant organizations, medical professionals, and local communities.

## Figures and Tables

**Table 1:** Characteristics of the participants according to the presence or absence of social isolation by sex (*n* = 22,933).

	Men (*n* = 8,059)			Women (*n* = 14,876)		
	Non-Social Isolation (*n* = 5652)	Social Isolation (*n* = 2407)	*p* Value	Non-Social Isolation (*n* = 10,926)	Social Isolation (*n* = 3948)	*p* Value
Age (SD)	60.6 (11.8)	58.9 (11.0)	<0.001	58.7 (12.0)	56.2 (11.4)	<0.001
Area (Coast, %)	75.3	73.6	0.104	65.9	62.1	<0.001
House damage due to the GEJE (%)	31.8	30.1	0.125	31.2	29.1	0.014
Death of family members due to the GEJE (%)	26.6	23.1	0.001	30.3	26.2	<0.001
Education level (%)						
Junior high school	26.0	24.9	0.677	22.7	21.7	0.258
High school	46.4	46.9		44.5	46.1	
College, university, and higher	26.7	27.2		31.9	31.2	
Other	0.9	1.0		0.9	1.0	
No job (%)	32.7	38.6	<0.001	49.3	46.5	0.003
Being married (%)	83.8	68.7	<0.001	76.8	71.8	<0.001
Living alone (%)	6.3	12.0	<0.001	8.6	9.0	0.477
Current smoker (%)	29.8	27.7	0.058	6.1	8.7	<0.001
Current drinker (%)	77.1	70.1	<0.001	34.2	33.6	0.484
Exercise habits (%)	44.0	36.6	<0.001	43.7	34.3	<0.001
BMI (%)						
<18.5 kg/m^2^	1.6	2.3	0.075	7.2	9.6	<0.001
18.5 to 24.9 kg/m^2^	62.8	63.3		65.7	65.2	
≥25.0 kg/m^2^	35.5	34.4		27.1	25.1	
Hypertension	33.3	32.1	0.271	24.5	20.2	<0.001
Diabetes mellitus	9.8	10.1	0.710	4.6	4.3	0.525
Hyperlipidemia	10.5	12.6	0.005	15.8	14.0	0.007
Depressive symptoms (CES-D ≥16, %)	16.0	32.2	<0.001	24.9	42.9	<0.001
Insomnia (AIS ≥6, %)	14.6	26.1	<0.001	22.5	34.6	<0.001
Lack of social capital (SC <9, %)	0.7	3.9	<0.001	0.6	3.3	<0.001

Social isolation: LSNS-6 <12; depressive symptoms: CES-D ≥16; insomnia: AIS ≥6; social capital: SC <9. LSNS-6, Lubben Social Network Scale-6; GEJE, Great East Japan Earthquake; BMI, body mass index; CES-D, Center for Epidemiologic Studies Depression Scale; AIS, Athene Insomnia Scale; SC, social capital. Statistical significance, *p* < 0.05.

**Table 2:** Adjusted HRs (95% CI) of total mortality according to social isolation by sex.

	Men (*n* = 8059)				
	No. of Participants	Observational Person-Years	No. of Deaths	HR (95% CI)	*p* Value
Non-social isolation	5652	28,440	145	1.00	
Social isolation	2407	11,994	83	1.38 (1.04–1.83)	0.027
	**Women (*n* = 14,874)**				
	**No. of participants**	**Observational person-years**	**No. of death** **s**	**HR (95% CI)**	***p*** **value**
Non-social isolation	10,926	55,288	93	1.00	
Social isolation	3948	19,902	40	1.49 (1.02–2.19)	0.040

HR, hazard ratio; 95% CI, 95% confidence interval. Social isolation, LSNS-6 <12. Adjusted for age, area, education level, working status, marital status, number of household members, smoking habits, drinking habits, exercise habits, BMI, medical history, depressive symptoms, AIS, SC, house damage due to the GEJE, and death of family members due to the GEJE. Statistical significance, *p* < 0.05.

**Table 3:** Adjusted HRs (95% CIs) of total mortality according to social isolation by age group.

	Men (*n* = 8059)					
	No. of Participants	Observational Person-Years	No. of Deaths	HR (95% CI)	*p* Value	*p* for Interaction
Age <65 (*n* = 4427)						
Non-social isolation	2914	14,412	46	1.00		0.660
Social isolation	1513	7511	37	1.26 (0.79–1.99)	0.332	
Age ≥65 (*n* = 3632)						
Non-social isolation	2738	14,028	99	1.00		
Social isolation	894	4483	46	1.45 (1.01–2.07)	0.045	
	**Women (*n* = 14,874)**					
	**No. of participants**	**Observational person-years**	**No. of deaths**	**HR (95% CI)**	***p* value**	***p*** **for** **Interaction**
Age <65 (*n* = 9510)						
Non-social isolation	6606	33,018	33	1.00		0.416
Social isolation	2904	14,565	18	1.16 (0.64–2.10)	0.618	
Age ≥65 (*n* = 5,364)						
Non-social isolation	4320	22,270	60	1.00		
Social isolation	1044	5337	22	1.73 (1.05–2.85)	0.032	

HR, hazard ratio; 95% CI, 95% confidence interval. Social isolation, LSNS-6 <12. Adjusted for age, area, education level, working status, marital status, number of household members, smoking habits, drinking habits, exercise habits, BMI, medical history, depressive symptoms, AIS, SC, house damage due to the GEJE, and death of family members due to the GEJE. Statistical significance, *p* < 0.05.

**Table 4:** Adjusted HRs (95% CI) of mortality according to house damage and social isolation.

	Men (*n* = 8059)					
House damage × Social isolation	No. of participants	Observational person-years	No. of deaths	HR (95% CI)	*p* value	*p* for Interaction
Undamaged × Non-social isolation	3854	19,468	95	1.00		0.153
Damaged × Non-social isolation	1798	8972	50	1.08 (0.76–1.54)	0.678	
Undamaged × Social isolation	1683	8420	60	1.46 (1.04–2.03)	0.027	
Damaged × Social isolation	724	3574	23	1.30 (0.81–2.10)	0.278	
	Women (*n* = 14,874)					
House damage × Social isolation	No. of participants	Observational person-years	No. of deaths	HR (95% CI)	*p* value	*p* for Interaction
Undamaged × Non-social isolation	7518	37,177	58	1.00		0.072
Damaged × Non-social isolation	3408	17,111	35	1.34 (0.87–2.07)	0.184	
Undamaged × Social isolation	2800	14,182	25	1.43 (0.89–2.30)	0.145	
Damaged × Social isolation	1148	5720	15	2.12 (1.17–3.83)	0.013	

HR, hazard ratio; 95% CI, 95% confidence interval. Social isolation, LSNS-6 <12. Adjusted for age, area, education level, working status, marital status, number of household members, smoking habits, drinking habits, exercise habits, BMI, medical history, depressive symptoms, AIS, SC, and death of family members due to the GEJE. Statistical significance, *p* < 0.05.

**Table 5:** Adjusted HRs (95% CI) of mortality according to the death of family members due to the GEJE and social isolation.

	Men (*n* = 8059)					
Death of family members due to the GEJE × Social isolation	No. of participants	Observational person-years	No. of deaths	HR (95% CI)	*p* value	*p* for Interaction
No death of family members due to the GEJE × Non-social isolation	4149	20,974	105	1.00		0.080
Death of family members due to the GEJE × Non-social isolation	1503	7466	40	0.94 (0.65–1.38)	0.763	
No death of family members due to the GEJE × Social isolation	1852	9260	58	1.24 (0.89–1.73)	0.202	
Death of family members due to the GEJE × Social isolation	555	2734	25	1.69 (1.07–2.66)	0.025	
	Women (*n* = 14,874)					
Death of family members due to the GEJE × Social isolation	No. of participants	Observational person-years	No. of deaths	HR (95% CI)	*p* value	*p* for Interaction
No death of family members due to the GEJE × Non-social isolation	7619	38,699	64	1.00		0.125
Death of family members due to the GEJE × Non-social isolation	3307	16589	29	0.89 (0.56–1.40)	0.611	
No death of family members due to the GEJE × Social isolation	2914	14754	31	1.60 (1.03–2.48)	0.037	
Death of family members due to the GEJE × Social isolation	1034	5148	9	1.06 (0.52–2.17)	0.871	

HR, hazard ratio; 95% CI, 95% confidence interval. Social isolation, LSNS-6 <12. Adjusted for age, area, education level, working status, marital status, number of household members, smoking habits, drinking habits, exercise habits, BMI, medical history, depressive symptoms, AIS, SC, and house damage due to the GEJE. Statistical significance, *p* < 0.05.

## Data Availability

Data sharing is not applicable to this article because of privacy or ethical restrictions.

## Supplementary Materials

The following supporting information can be downloaded at: https://www.mdpi.com/article/10.3390/ijerph19074343/s1, Table S1: Adjusted HRs (95% CI) of total mortality according to social isolation by sex excluding participants who died within 1 year after baseline survey (*n* = 22,892); Table S2: Adjusted HRs (95% CIs) of total mortality according to social isolation by age group excluding participants who died within 1 year after baseline survey (*n* = 22,892); Table S3: Adjusted HRs (95% CI) of mortality according to house damage and social isolation excluding participants who died within 1 year after baseline survey (*n* = 22,892); Table S4: Adjusted HRs (95% CI) of mortality according to the death of family members due to the GEJE and social isolation excluding participants who died within 1 year after baseline survey (*n* = 22,892).
