# Supplementary material for: Association between Social Isolation and Total Mortality after the Great East Japan Earthquake in Iwate Prefecture: Findings from the TMM CommCohort Study
Source: Int J Environ Res Public Health. 2022 Apr 5;19(7):4343. doi: 10.3390/ijerph19074343 (PMC8998212; doi:10.3390/ijerph19074343)
Supplement: Supplementary file 1 [file ijerph-19-04343-s001.zip › ijerph-1640111-supplementary.pdf]

Supplementary Materials

Table S1. Adjusted HRs (95% CI) of total mortality according to social isolation by sex excluding participants who died within 1 year after baseline survey (n = 22,892).

	Men (n = 8,028)				
	No. of participants	Observational person-years	No. of deaths	HR (95% CI)	*P* value
Non-social isolation	5,635	28,430	128	1.00	
Social isolation	2,393	11,985	69	1.29 (0.95 – 1.75)	0.106
	Women (n = 14,864)				
	No. of participants	Observational person-years	No. of deaths	HR (95% CI)	*P* value
Non-social isolation	10,919	55,284	86	1.00	
Social isolation	3,945	19,900	37	1.50 (1.01 – 2.23)	0.046

HR, hazard ratio; 95% CI, 95% confidence interval.
Social isolation, LSNS-6 < 12.
Adjusted for age, area, education level, working status, marital status, number of household members, smoking habits, drinking habits, exercise habits, BMI, medical history, depressive symptoms, AIS, SC, house damage due to the GEJE, death of family members due to the GEJE.
Statistical significance, P < 0.05.

Table S2. Adjusted HRs (95% CIs) of total mortality according to social isolation by age group excluding participants who died within 1 year after baseline survey (n = 22,892).

	Men (n = 8,028)					
	No. of participants	Observational person-years	No. of deaths	HR (95% CI)	*P* value	*P* for Interaction
Age <65 (n = 4,410)						
Non-social isolation	2,907	14,408	39	1.00		0.292
Social isolation	1,503	7,503	27	1.00 (0.59 – 1.68)	0.985	
Age ≥65 (n = 3,618)						
Non-social isolation	2,728	14,023	89	1.00		
Social isolation	890	4,481	42	1.45 (1.00 – 2.12)	0.054	
	**Women (n = 14,864)**					
	No. of participants	Observational person-years	No. of deaths	HR (95% CI)	*P* value	*P* for Interaction
Age <65 (n = 9,505)						
Non-social isolation	6,603	33,016	30	1.00		0.385
Social isolation	2,902	14,564	16	1.16 (0.62 – 2.17)	0.645	
Age ≥65 (n = 5,359)						
Non-social isolation	4,316	22,268	56	1.00		
Social isolation	1,043	5,337	21	1.77 (1.06 – 2.96)	0.029	

HR, hazard ratio; 95% CI, 95% confidence interval.
Social isolation, LSNS-6 < 12.
Adjusted for age, area, education level, working status, marital status, number of household members, smoking habits, drinking habits, exercise habits, BMI, medical history, depressive symptoms, AIS, SC, house damage due to the GEJE, death of family members due to the GEJE.
Statistical significance, $P < 0.05$.

Table S3. Adjusted HRs (95% CI) of mortality according to house damage and social isolation excluding participants who died within 1 year after baseline survey (n = 22,892).

	Men (n = 8,028)					
House damage × Social isolation	No. of participants	Observational person-years	No. of deaths	HR (95% CI)	*P* value	*P* for Interaction
Undamaged × Non-social isolation	3,842	19,462	83	1.00		0.331
Damaged × Non-social isolation	1,793	8,969	45	1.11 (0.77 – 1.62)	0.572	
Undamaged × Social isolation	1,674	8,414	51	1.40 (0.98 – 2.01)	0.065	
Damaged × Social isolation	719	3,570	18	1.17 (0.69 – 1.99)	0.562	
	Women (n = 14,864)					
House damage × Social isolation	No. of participants	Observational person-years	No. of deaths	HR (95% CI)	*P* value	*P* for Interaction
Undamaged × Non-social isolation	7,513	38,174	53	1.00		0.048
Damaged × Non-social isolation	3,406	17,110	33	1.44 (0.92 – 2.26)	0.112	
Undamaged × Social isolation	2,798	14,181	23	1.45 (0.88 – 2.39)	0.142	
Damaged × Social isolation	1,147	5,719	14	2.28 (1.23 – 4.21)	0.008	

HR, hazard ratio; 95% CI, 95% confidence interval.
Social isolation, LSNS-6 < 12.
Adjusted for age, area, education level, working status, marital status, number of household members, smoking habits, drinking habits, exercise habits, BMI, medical history, depressive symptoms, AIS, SC, death of family members due to the GEJE.
Statistical significance, $P < 0.05$.

Table S4. Adjusted HRs (95% CI) of mortality according to the death of family members due to the GEJE and social isolation excluding participants who died within 1 year after baseline survey (n = 22,892).

	Men (n = 8,028)					
Death of family members due to the GEJE × Social isolation	No. of participants	Observational person-years	No. of deaths	HR (95% CI)	*P* value	*P* for Interaction
No death of family members due to the GEJE × Non-social isolation	4,136	20,967	92	1.00		0.309
Death of family members due to the GEJE × Non-social isolation	1,499	7,464	36	0.95 (0.64 – 1.42)	0.81	
No death of family members due to the GEJE ×Social isolation	1,843	9,255	49	1.19 (0.83 – 1.70)	0.351	
Death of family members due to the GEJE × Social isolation	550	2,730	20	1.52 (0.92 – 2.52)	0.105	
	Women (n = 14,864)					
Death of family members due to the GEJE × Social isolation	No. of participants	Observational person-years	No. of deaths	HR (95% CI)	*P* value	*P* for Interaction
No death of family members due to the GEJE × Non-social isolation	7,616	38,697	61	1.00		0.085
Death of family members due to the GEJE × Non-social isolation	3,303	16,587	25	0.79 (0.49 – 1.28)	0.333	
No death of family members due to the GEJE × Social isolation	2,912	14,753	29	1.59 (1.01 – 2.50)	0.045	
Death of family members due to the GEJE × Social isolation	1,033	5,147	8	0.99 (0.46 – 2.10)	0.974	

HR, hazard ratio; 95% CI, 95% confidence interval.
Social isolation, LSNS-6 < 12.
Adjusted for age, area, education level, working status, marital status, number of household members, smoking habits, drinking habits, exercise habits, BMI, medical history, depressive symptoms, AIS, SC, house damage due to the GEJE.
Statistical significance, P < 0.05.
